# Complete thrombosis of a bioprosthetic mitral valve and left atrium in a patient on venoarterial extracorporeal membrane oxygenation with favorable outcome

**DOI:** 10.1186/s13019-024-02916-3

**Published:** 2024-06-26

**Authors:** James Hall, Michael Khilkin, Sunil Abrol

**Affiliations:** Department of Cardiac Surgery, New York University Langone Health, NYU Long Island Hospital, 259 First St, Mineola, New York, 11501 USA

## Abstract

Intra-cardiac thrombosis is a potentially devastating complication of extracorporeal membrane oxygenation (ECMO) mechanical circulatory support. We present here a patient who suffered complete thrombosis of a fresh mitral prosthesis and left atrium in the setting of ECMO with aortic insufficiency who was treated with repeat valve replacement and thrombectomy. To our knowledge, she is the only patient in the reported literature to have survived this complication.

## Case presentation

A seventy year old female with a past medical history of hypertension, dyslipidemia, and anxiety presented with malaise, night sweats, shortness of breath, nausea, and a right hand partial paresis and was found to have blood cultures positive for *streptococcus bovis.* MRI identified three small subacute strokes, and subsequent transesophageal echocardiography identified a mobile filamentous structure on her mitral valve, mild to moderate aortic valve insufficiency, and a patent foramen ovale. She was treated with ceftriaxone and referred for mitral valve replacement.

After one day of antibiotics, she developed respiratory distress and presented with progressive hypotension, lactic acidosis, and shortness of breath with moderate pulmonary vascular congestion. Repeat echocardiography showed severe mitral regurgitation with an 11 mm x 2 mm mobile arcuate density tethered to the anterior mitral leaflet, a left ventricular (LV) ejection fraction of 77%, and moderate aortic valve insufficiency. Her right ventricular function was normal. The antibiotics were continued and she was treated with diuretics in preparation for surgery.

**Fig. 1:**
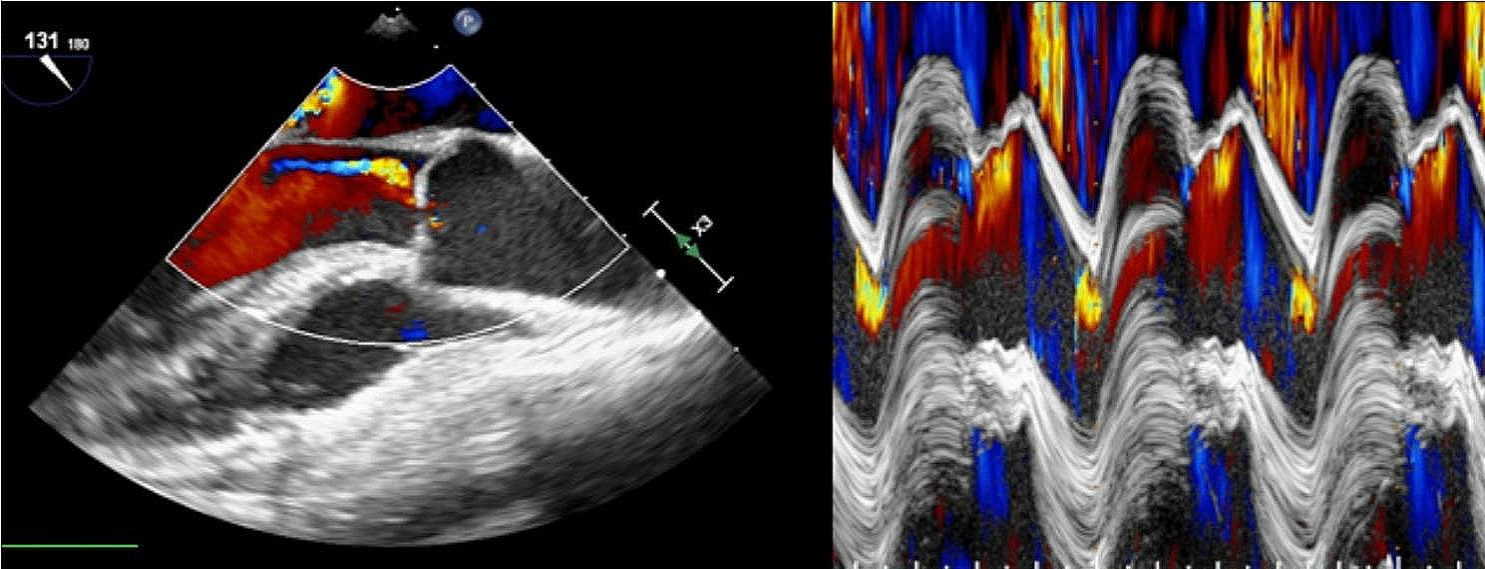
Color doppler 2D and color enhanced M-mode images from intraoperative transesophageal echocardiography with IABP in place

Cardiac catheterization showed no significant coronary disease and significantly less aortic insufficiency on ventriculogram, so an intra-aortic balloon pump (IABP) was placed for severe mitral regurgitation. However, despite aggressive diuresis, her pulmonary congestion worsened and her cardiac index dropped to 1.37. She was taken the following morning to the operating room (OR) and found to have mitral vegetations and several ruptured chordae. A chordae sparing mitral valve replacement (27 mm Mitris Resilia Valve, Edwards Life Sciences, Irvine, California) was performed. While in the OR, she experienced significant right heart dysfunction, and peripheral venoarterial extracorporeal membrane oxygenation (ECMO) through a femoral vein and artery was initiated. The aortic insufficiency was judged to be mild with the IABP running, so it was left in place for afterload reduction [Figure 1]. Limited postoperative echocardiography showed an ejection fraction of 20%, right ventricular failure with a tricuspid annular planar systolic excursion (TAPSE) of 0.5 cm, and moderate aortic insufficiency. Postoperatively on day (POD) 0, she developed a worsened lactic acidosis and her IABP was replaced with an Impella CP device (Abiomed, Danvers Massachusetts) resulting in apparent improvement of both right and left ventricular function.

**Fig. 2:**
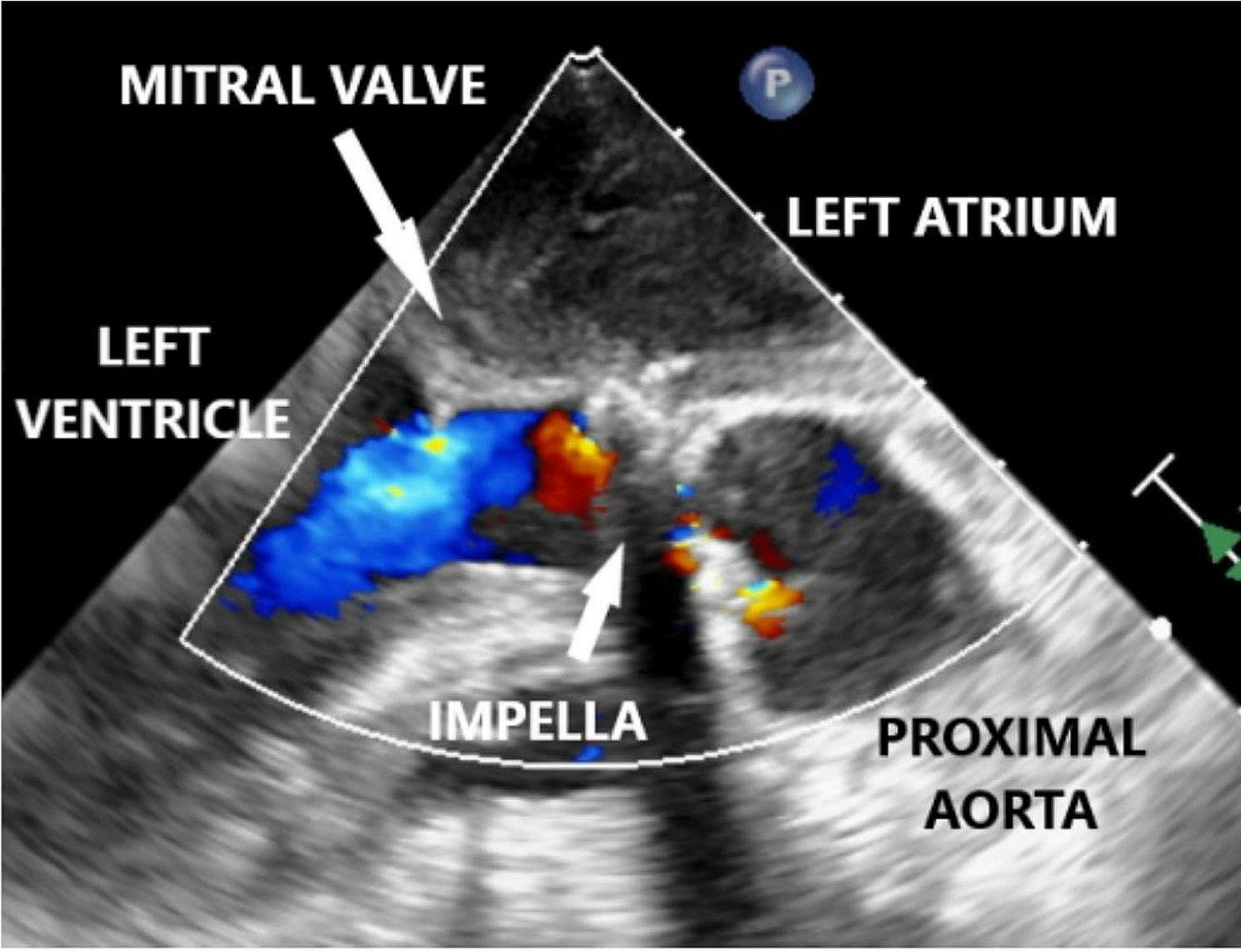
Transesophageal echocardiogram, midesophageal aortic valve long-axis view showing the left atrium with spontaneous echo contrast, a thickened, non-functional mitral valve, the left ventricle with an aortic regurgitant jet, the Impella as it passes through the aortic valve with a large shadowing artifact below it, and the proximal ascending aorta

**Fig. 3:**
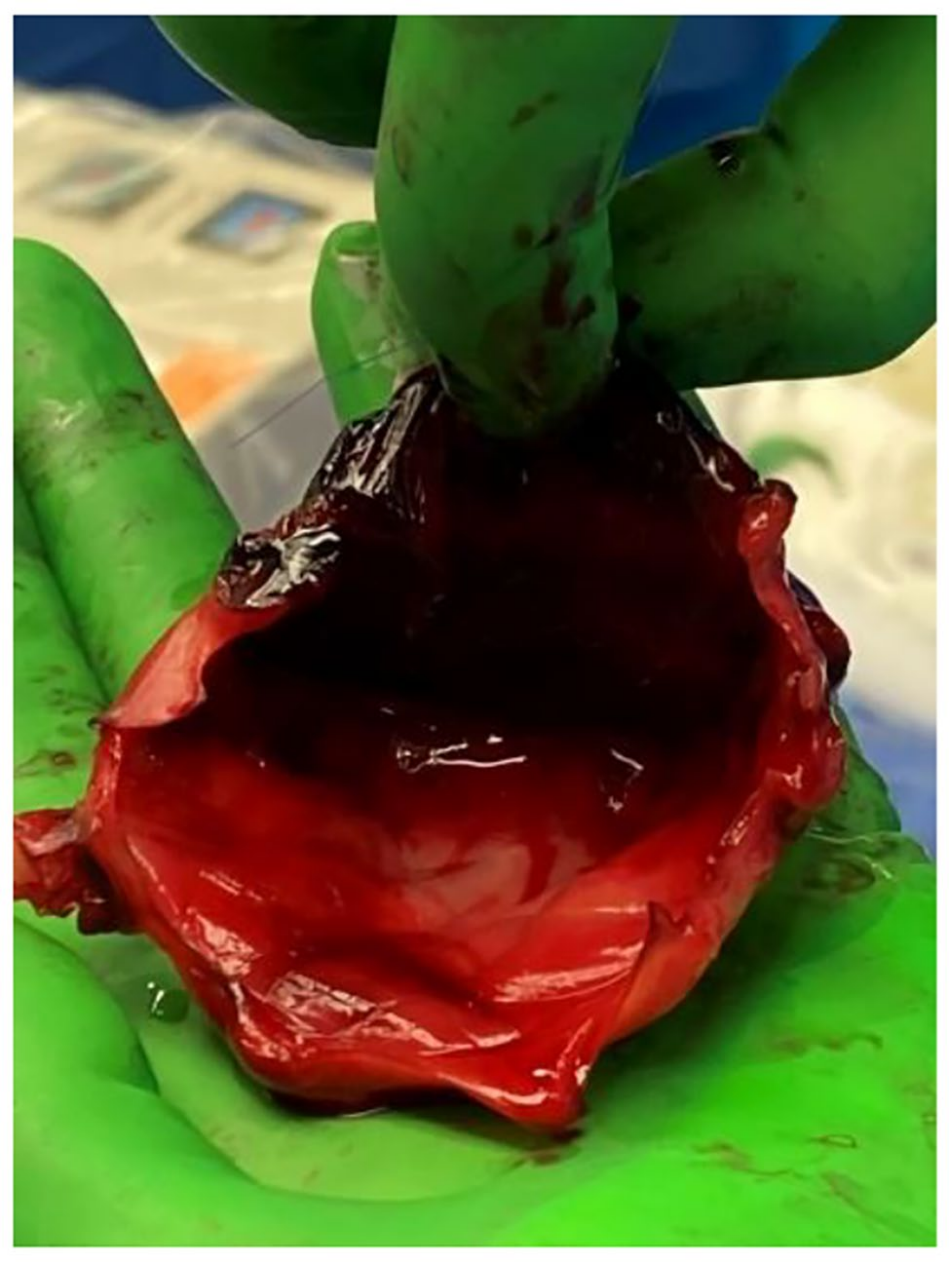
Laminar cast thrombosis of the left atrium removed during reoperation

**Fig. 4:**
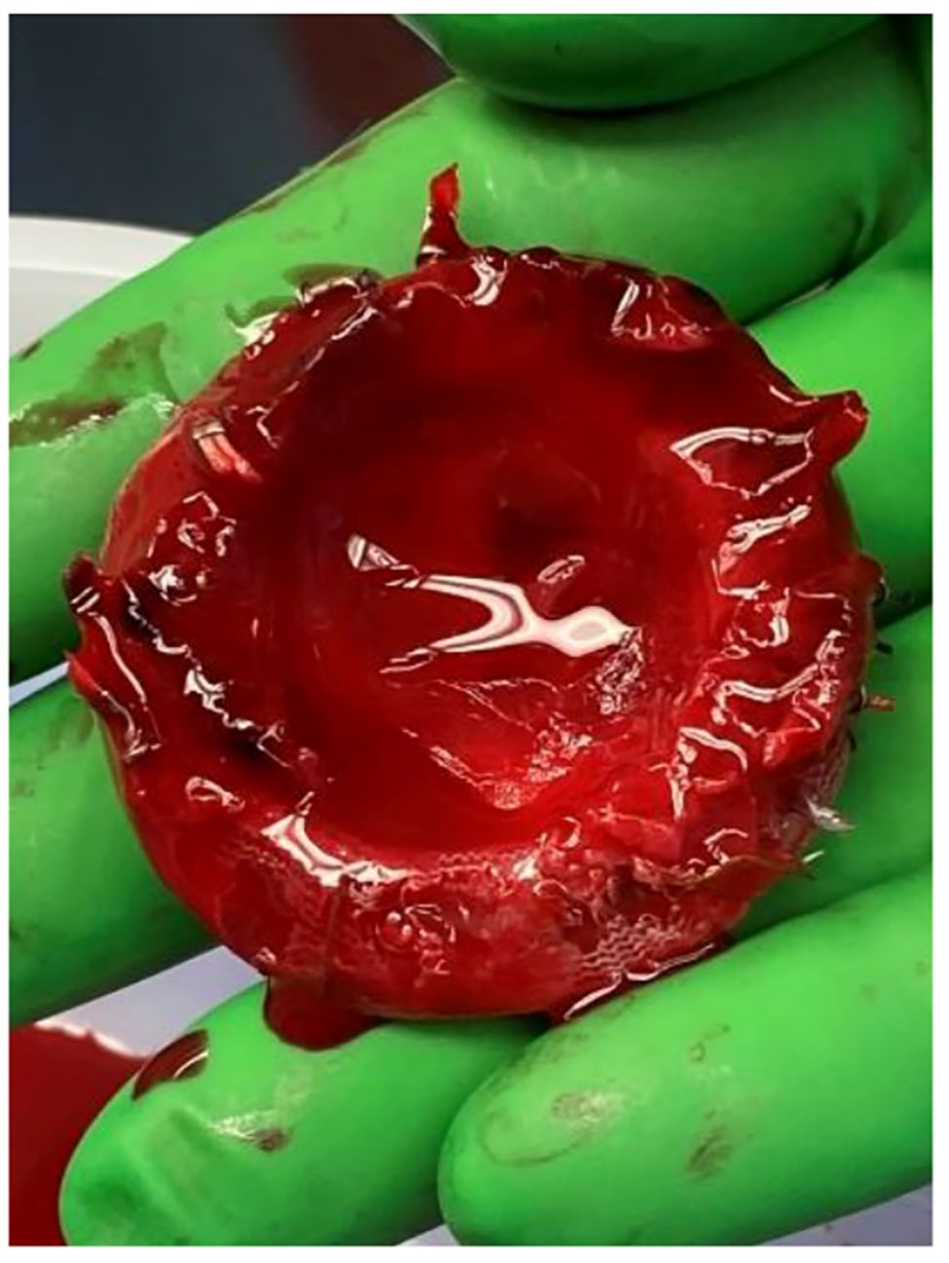
Bioprosthetic mitral valve coated in laminar thrombosis

On POD 1, the patient developed hemoptysis, increasing pulmonary pressures, and a brief episode of ventricular tachycardia. Bronchoscopy showed no clear source of bleeding. Over PODs 2–3, echocardiography showed severely dilated left and right ventricles and continued aortic regurgitation. Increasing Impella power consistently resulted in suction alarms, and attempts to reduce ECMO support resulted in immediate hypotension. She required initiation of continuous renal replacement therapy. On POD 4, she was taken back to the OR for Impella removal where she was found to have a completely thrombosed left atrium extending into all four pulmonary veins and a prosthetic mitral valve coated with laminated mature clot [Figs. 2, 3, 4]. Between the operations, the patient was thrombocytopenic with platelet counts from 33 to 56 10³/uL and on a heparin drip at 12 U/kg/hr. Her ECMO flows were maintained at 2.5–3 L/min for a cardiac index of > 2.0. Lactate levels remained normal except for small increases during ECMO weaning attempts.

No clot was found in her left ventricle. She was found to have a torn cusp of her aortic valve. The atrial clot was removed and the atria and pulmonary veins were irrigated aggressively. The thrombosed valve was replaced with a 25 mm epic™ porcine valve (Abbott Laboratories, Chicago, Illinois), and the aortic valve was replaced with a 19 mm bovine tissue valve.

The Impella was removed and her chest was left open while ECMO support continued.

Over POD 5–6, she was maintained on inotropes and nitric oxide and diuresed. Her contractility improved, and on POD 7, she returned to the OR for chest closure. On POD 9, she was weaned from ECMO and decannulated. She was extubated on POD 11 with excellent mental status and diuresed well with improving renal function. Over the following days, she was weaned from inotropic support and a biventricular pacing defibrillator (Boston Scientific, Marlborough, Massachusetts) was placed for an intermittent heart block.

By POD 21, her LV ejection fraction was 55% with mild right ventricular hypokinesis. She was discharged and at a two week follow up appointment was walking a mile per day with no further sequelae.

## Discussion

Venoarterial (VA) extracorporeal membrane oxygenation is a life-saving intervention for acute cardiovascular collapse due to cardiogenic and obstructive causes. Nonetheless, the alterations in flow dynamics created by the peripheral configuration creates the conditions for significant complications, mostly related to a large increase in afterload on an already weakened left ventricle. This increase in afterload can reduce the trans-aortic valve gradient to the point that multiple ventricular contractions may be required to achieve pressures sufficient for ejection with resultant dilation of the ventricle in accordance with the Frank-Starling principle. This can lead to relative stasis of the blood, increasing the risk for intracardiac thrombosis as well as further weakening of contractions and increased arrhythmias from LV dilation. This can be managed by venting the LV using a variety of techniques including atrial septostomy, LV drain, and percutaneous axial pumps [1].

In this setting, blood clots remain a relatively uncommon occurrence, but can happen regardless of anticoagulation. One retrospective cohort study showed a rate of 3.9%, carrying a mortality of 100% [2]. Another case review identified 12 patients with intracardiac thrombosis, most of whom had clots in multiple chambers, 10 of whom died. The same review showed that as many as half the patients who died while on VA ECMO had evidence on autopsy of intracardiac or systemic thrombosis suggesting under recognition or under reporting [3]. In addition, while far less common than with mechanical valves, bioprosthetic valves do have an inherent risk of thrombosis reported at 0.74% per year with the tricuspid valve the most commonly affected [2].

Both the precipitating disease process and the exposure of blood to the ECMO circuit components induce a proinflammatory state and increase the risk for clot formation [4]. Additionally, the flow disruptions created by the increased afterload in the system promote stasis and even flow reversal [5]. The most common locations for thrombus are the LV cavity and the proximal ascending aorta, particularly if LV ejection is impaired. This can be addressed by LV venting or reducing ECMO circuit flows, if tolerated. Another complicating factor is pre-existent or exacerbated valvulopathy. Stenotic valves add to the already increased afterload, and regurgitant valves, particularly the aortic valve, create a situation requiring duplication of work to maintain forward flow given the recirculated volumes. For this reason, severe aortic insufficiency is listed as a strong relative contraindication to VA ECMO, described as ‘almost immediately prohibitive’ [6]. Nonetheless, VA ECMO has been used to successfully treat severe aortic insufficiency if used as a bridge to immediate surgery or with the use of a biatrial drain inserted venously, but placed across the intra-atrial septum to drain both atria simultaneously [7, 8].

In our patient, it appears that the recirculation of blood between the left ventricle and proximal aorta created by the Impella and aortic regurgitation allowed diastolic pressures in the LV to exceed those in the left atrium during atrial systole thereby preventing valve opening and anterograde filling. This likely caused the laminar clot to form on the bioprosthetic valve and resulted in the classic symptoms of acute mitral stenosis, specifically pulmonary edema, right ventricular failure, hemoptysis, and pulmonary hypertension [9]. Such an account would also explain her complete dependence on the ECMO circuit and the inability to increase flows through the Impella.

Another case reported in the literature bears some similarities. In that case, a patient on VA ECMO was vented via septostomy at a sufficient volume to divert nearly all flow into a left to right shunt, resulting in a similar bioprosthetic thrombosis. This patient did not clot her atrium, but did also required excision and replacement of the prosthetic valve. She survived to decannulation, but not to discharge [10].

Three prior case reports were identified with left atrial thrombi on VA ECMO, and all three expired during treatment [11–13]. To our knowledge, this is the only case to be reported of a patient not only surviving left atrial thrombus, but going on to full recovery.

## Data Availability

No datasets were generated or analysed during the current study.
